# Tuning of Magnetic Damping in Y_3_Fe_5_O_12_/Metal Bilayers for Spin-Wave Conduit Termination

**DOI:** 10.3390/ma15082814

**Published:** 2022-04-12

**Authors:** Adam Krysztofik, Nikolai Kuznetsov, Huajun Qin, Lukáš Flajšman, Emerson Coy, Sebastiaan van Dijken

**Affiliations:** 1 Institute of Molecular Physics, Polish Academy of Sciences, Smoluchowskiego 17, 60-179 Poznan, Poland; 2 NanoSpin, Department of Applied Physics, Aalto University School of Science, P.O. Box 15100, FI-00076 Aalto, Finland; nikolai.1.kuznetsov@aalto.fi (N.K.); huajun.qin@aalto.fi (H.Q.); lukas.flajsman@aalto.fi (L.F.); sebastiaan.van.dijken@aalto.fi (S.v.D.); 3 NanoBioMedical Centre, Adam Mickiewicz University, Wszechnicy Piastowskiej 3, 61-614 Poznan, Poland; coyeme@amu.edu.pl

**Keywords:** yttrium iron garnet, YIG, ferromagnetic resonance, effective damping parameter, spin-waves, spin wave packet

## Abstract

In this work, we investigate the structural and dynamic magnetic properties of yttrium iron garnet (YIG) films grown onto gadolinium gallium garnet (GGG) substrates with thin platinum, iridium, and gold spacer layers. Separation of the YIG film from the GGG substrate by a metal film strongly affects the crystalline structure of YIG and its magnetic damping. Despite the presence of structural defects, however, the YIG films exhibit a clear ferromagnetic resonance response. The ability to tune the magnetic damping without substantial changes to magnetization offers attractive prospects for the design of complex spin-wave conduits. We show that the insertion of a 1-nm-thick metal layer between YIG and GGG already increases the effective damping parameter enough to efficiently absorb spin waves. This bilayer structure can therefore be utilized for magnonic waveguide termination. Investigating the dispersionless propagation of spin-wave packets, we demonstrate that a damping unit consisting of the YIG/metal bilayers can dissipate incident spin-wave signals with reflection coefficient *R* < 0.1 at a distance comparable to the spatial width of the wave packet.

## 1. Introduction

The employment of spin-waves (SW) for performing logic operations is considered to be an innovative concept competing with the standard CMOS paradigm [1,2,3,4,5,6,7]. A material of choice for complex SW conduits is yttrium iron garnet (YIG) in the single-crystal phase [8]. The growth of ultra-low damping YIG films has been optimized in the last decade [9,10,11,12] and the development of garnet layers is ongoing [13,14,15,16,17,18,19,20,21,22,23,24,25,26]. Hitherto, state-of-art YIG films provide high relaxation times of hundreds of nanoseconds corresponding to millimeter-scale SW decay length [27,28,29]. These long propagation distances pose questions on how to attenuate SWs when waveguides terminate without causing back reflection and signal interference. In micromagnetic simulations, this problem is solved by applying parabolic or exponential damping conditions near the edges of magnetic structures [30,31,32,33,34]. However, the experimental realization of this approach is not trivial. Up to now, the standard experimental approach has involved the use of elongated SW waveguides, usually with canted ends [27,28,35,36]. Moreover, a recent study showed that the SW intensity is well maintained in nanoscopic, tapered waveguides [37]. Following further miniaturization and an increased packing of SW devices, controlled SW damping will become important in limiting back reflection at open-ended or unused waveguide ports.

In the current paper, we propose the utilization of YIG/metal bilayers for SW conduit termination. We systematically investigate the magnetic properties of crystallized YIG films on GGG substrates with wedge-shaped metal films. For this study, three consecutive metals in the periodic table were chosen, namely Ir, Pt, and Au, which are resistant to oxidation. This article is organized as follows. In Section 2, we describe the growth methods, sample preparation, and the used experimental apparatus. In Section 3.1 and Section 3.2, we present the structural and magnetic properties of the YIG films. In Section 3.3, we analyze the propagation of SW packets in continuous YIG films. In Section 3.4, we investigate SW packet reflection from a damping unit using micromagnetic simulations. Section 4 summarizes the study.

## 2. Materials and Methods

We use the pulsed laser deposition (PLD) technique to grow 40-nm-thick YIG films (Nd:YAG laser, 355 nm) and magnetron sputtering to deposit thin metal layers. The growth processes were conducted at room temperature for both methods. The GGG substrates were ultrasonicated in acetone and isopropanol before loading into the deposition chamber. Wedge-shaped layers of Pt, Ir, and Au were deposited with a thickness increase of 0.5 nm per millimeter by moving a shutter plate at a constant speed over the sample surface (2 cm × 0.5 cm) during the deposition process. This resulted in 0–7 nm wedge films and an uncovered substrate area (6 mm in length), which we used as a reference. Subsequently, the samples were transferred to the PLD chamber without breaking the vacuum. The target-to-substrate distance was 5 cm. The pulse frequency of 2 Hz yielded a growth rate of ≈0.65 nm/min at the partial oxygen pressure of 2.4 × 10^−2^ Pa (2.4 × 10^−4^ mbar) [38]. After the deposition, the bilayers were annealed ex-situ in air for 5 min at 800 °C. For VNA spectroscopy measurements, 150-nm-thick Au microwave antennas with a width of 2 μm were fabricated using direct laser-writing lithography (Laserwriter LW 405), magnetron sputtering, and lift-off.

The structural properties of the films were investigated using X-ray diffraction and grazing incidence X-ray diffraction (Seifert system 3003TT) as well as high-resolution XRD utilizing a four-crystal Ge (220) monochromator (Malvern Panalytical, Malvern, UK, X’pert Pro3 system). The scans provided the out-of-plane lattice parameters. Nominal values of film thicknesses were confirmed with X-ray reflectivity (XRR) measurements (Seifert system 3003TT). The surface topography was investigated with atomic force microscopy (AFM) using a Bruker ICON microscope and scanning electron microscopy (SEM) using FEI Nova NanoSEM 650 with a concentric back-scattered (CBS) detector. From the contrast changes in SEM images, the height-height correlation function (HHCF) was evaluated to estimate the lateral correlation length of a defect [39]. The dynamic magnetic properties were studied with a broadband ferromagnetic resonance setup in field-sweep mode (VNA-FMR), with VNA spectroscopy in frequency-sweep mode utilizing a set of microwave antennas, as well as with super-Nyquist-sampling magneto-optical Kerr effect microscopy (SNS-MOKE) [40]. All measurements were performed at room temperature with an in-plane applied magnetic field. The determined uncertainties are one standard deviation unless noted otherwise. To investigate SW packet propagation, micromagnetic simulations were performed using open-source GPU-accelerated *MuMax3* software [41].

## 3. Results and Discussions

### 3.1. Structural Properties

**Figure 1:**
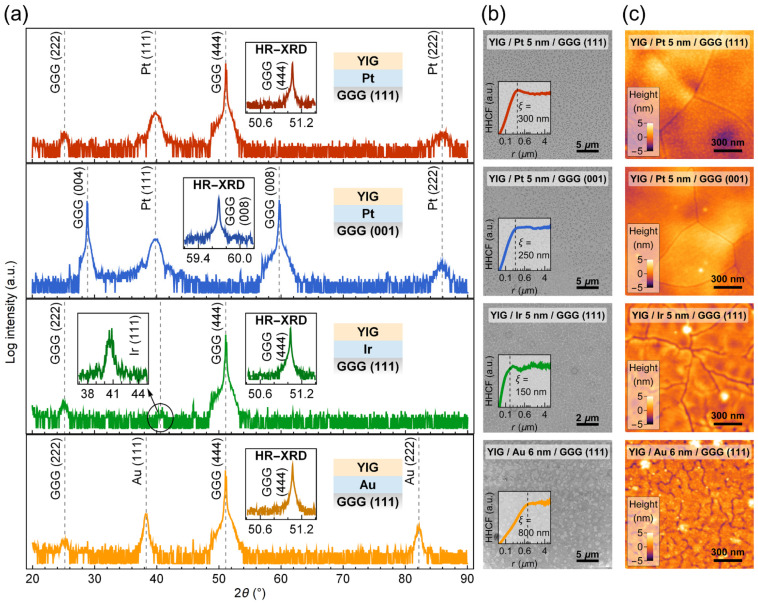
Structural properties of 40-nm-thick YIG films grown on GGG substrates with thin metal layers. (**a**) X-ray diffraction patterns recorded on samples with 5–7 nm metal underlayers. Note that the (222) peaks of the GGG substrate are so-called basis-forbidden reflections due to multiple diffraction [43]. Insets denoted as HR-XRD show high-resolution scans utilizing a four-crystal monochromator. The inset showing the Ir (111) reflection comes from a separate scan with a long statistical exposure. (**b**) SEM surface images. Insets show height-height correlation function (HHCF) as a function of lateral distance r calculated on the basis of SEM contrast changes to evaluate the defect correlation length ξ. (**c**) AFM topography maps.

X-ray diffraction patterns (Figure 1a) display a lack of YIG reflections if the YIG film is grown onto Pt, Ir, or Au. For coarse, polycrystalline YIG samples, XRD reflections are expected at 32.326° ((024), 100%), 35.510° ((224), 43%), or 28.835° ((004), 32%) [42].

High-resolution scans around the main reflection of the single-crystal GGG substrate (insets in Figure 1a) further show that the YIG films are not characterized by a strong texture. Similarly to YIG layers deposited on silicon [44], this suggests that the films are nanocrystalline. The large intensity of Pt (111) and Au (111) reflections for 5-nm-thick layers as well as the absence of peaks from other family planes indicate that these metal films are textured. The much smaller intensity of the Ir (111) reflection, being nearly at the level of the background noise, demonstrates polycrystalline film growth of this metal on the GGG substrate. The determined lattice parameters of 0.392 nm for Pt on both GGG (111) and GGG (001), 0.384 nm for Ir, and 0.407 nm for Au are in agreement with the bulk values within a 1% error [45]. To further investigate the structural properties of YIG, we performed GI-XRD scans (see supplementary materials). Despite the small peak-to-background ratio, YIG reflections are clearly measured. From this, we estimated a mean crystallite size of 16.9 ± 2.7 nm corresponding to ≈13 YIG lattice constants. Additionally, XRR results confirm a nominal YIG film thickness of 40.2 ± 1.3 nm, point to a consistent film density in different samples, but with increased roughness.

SEM and AFM measurements show significant structural defects in the YIG films grown onto the metal layers. In SEM images of the samples (Figure 1b), clear variations are visible in mixed topographic and compositional contrast as recorded using a CBS detector. Differences in defect length scales and size dispersion are also noticeable as inferred by the height-height correlation function (insets in Figure 1b). More thorough information about the surface morphology of YIG is provided by AFM imaging (Figure 1c). For all samples, nanoscale cracking is present, which can be understood as resulting from different thermal expansion coefficients of the metals and the garnets. Interestingly, fracturing of the YIG film is more severe for the Au layer than for Ir or Pt layers of similar thickness. This can be attributed to the early stages of Au shrinking towards the formation of Au nanoparticles or nanorods during the 5 min thermal annealing step [46,47]. This interpretation is congruent with the island-like pattern seen in the SEM images, corresponding to a defect correlation length of 800 nm. The YIG films on Pt exhibit larger flat areas between cracks when compared to Ir or Au. However, we observe additional height variations of ≈5 nm that are most likely caused by inhomogeneous stress in the YIG/Pt system (see bright and dark contrast in Figure 1c). The origin of these deformations is tentatively attributed to the high ductility and malleability of Pt as well as a lower thermal expansion coefficient when compared to Au [48]. Both these factors play a role during the post-annealing of the YIG film for crystallization.

### 3.2. Magnetization Dynamics

**Figure 2:**
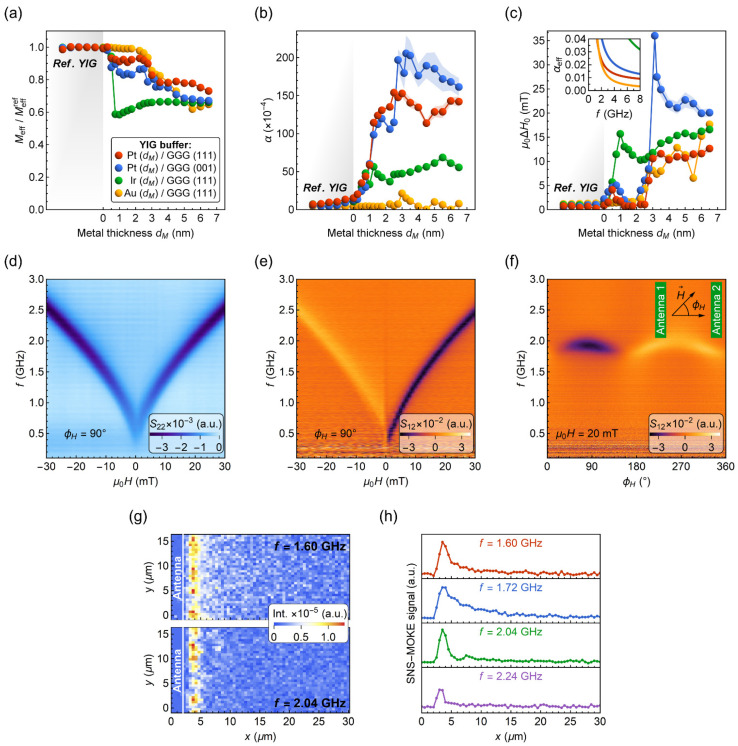
Broadband ferromagnetic resonance results for 40-nm-thick YIG films deposited on different metal underlayers with a nominal thickness $d_{m}$. (**a**) Effective magnetization $M_{\mathrm{eff}}$ normalized to $M_{\mathrm{eff}}^{\mathrm{ref}}$ of a reference YIG film. (**b**) Gilbert damping parameter α. (**c**) Inhomogeneous linewidth broadening $\mu_{0}\Delta H_{0}$. The inset shows calculated values of the effective damping parameter with Equation (3) for a 1-nm-thick metal layer. The color legend depicted in (**a**) also applies to (**b**,**c**). Values marked as reference YIG (shaded section) derive from measurements of the epitaxial film taken at the positions outside the metal wedge. (**d**–**f**) VNA spectroscopy results measured with lithographically patterned antennas for YIG (40 nm)/Au (3.0 nm)/GGG (111). (**d**) Color-coded reflection parameter S_22_ showing the FMR absorption. (**e**) Color-coded transmission parameter S_12_ for the magnetic field aligned parallel to the antennas ($\phi_{H}=$ 90°). (**f**) Color-coded angular dependence of S_12_ spectrum. Inset depicts in-plane magnetic field orientation with respect to the antenna geometry ($\phi_{H}=$ 0° for the magnetic field aligned perpendicular to the antenna edge). In figures (**d**–**f**), the real part of the scattering parameter S_pq_ is plotted. (**g**,**h**) SNS-MOKE microscopy maps and line profiles recorded at $\mu_{0}H=$ 20 mT for YIG (40 nm)/Au (3.0 nm)/GGG (111).

The insertion of a metal layer between a YIG film and a GGG drastically impacts the magnetization dynamics as found by broadband ferromagnetic resonance measurements. First, we analyze the relation between frequency f and resonance magnetic field H using the Kittel equation:

(1) $$f=\frac{\gamma \mu_{0}}{2\pi }\sqrt{H\left(H+M_{\mathrm{eff}}\right)},$$

where $M_{\mathrm{eff}}$ is the effective magnetization, γ is the gyromagnetic ratio, and $\mu_{0}$ is the vacuum permeability. Fitting experimental data to Equation (1) (see supplementary materials), we determine $M_{\mathrm{eff}}^{\mathrm{ref}}$ in the range of 115–190 kA/m for reference YIG films on GGG in agreement with the previous reports [8]. With an increasing metal layer thickness $d_{m}$, the value of $M_{\mathrm{eff}}$ decreases and it is reduced to 0.7 $M_{\mathrm{eff}}^{\mathrm{ref}}$ for $d_{m}=$ 6–7 nm (Figure 2a). For very thin metal layers ($d_{m}=$ 0.5–2.5 nm), however, no significant changes in $M_{\mathrm{eff}}$ are observed for Au, while for Pt/GGG (111) the decrease of $M_{\mathrm{eff}}$ is small (≈0.92 $M_{\mathrm{eff}}^{\mathrm{ref}}$). The $M_{\mathrm{eff}}$ of the YIG film deposited on Ir, on the other hand, diminishes rapidly at the onset of the metal wedge.

The parameters characterizing SW damping (Figure 2b,c), i.e., the Gilbert damping parameter α and the inhomogeneous linewidth broadening $\Delta H_{0}$ of the YIG films, were evaluated by measuring the dependence of the FMR linewidth ΔH on frequency f:

(2) $$\mu_{0}\Delta H=\alpha \frac{4\pi }{\gamma }f+\mu_{0}\Delta H_{0}.$$

At the onset of the Pt or Ir wedge layer, we observe a significant increase of the α parameter, which saturates for metal thicknesses $d_{m}>$ 2.5 nm at α≈ (150–180)·10^−4^ for Pt and α≈ (50–70)·10^−4^ for Ir. Considering the spin pumping effect, typical values of the spin mixing conductance vary between 5·10^17^ m^−2^ and 7·10^18^ m^−2^, which corresponds to an increase in the α parameter of Δα≈ (1–19)·10^−4^ for 40 nm thick YIG [49,50,51]. This shows that the damping-like torque arising from a nonequilibrium spin accumulation at the metal/YIG interface [52] contributes only weakly to the overall intrinsic damping in the samples [53,54].

Considering the excitation of microwave eddy currents in metal layers, we find this damping contribution to the α parameter to be less than 1·10^−4^ for 5-nm-thick metal films, and thus negligible [53,55,56]. Therefore, we attribute the increase of α to a deterioration of the structural properties when YIG is grown onto Pt or Ir. Surprisingly, the Au layer does not deteriorate the α parameter (≈8·10^−4^) for the entire thickness range. Moreover, as shown in Figure 2c, the inhomogeneous linewidth broadening $\mu_{0}\Delta H_{0}$ of ≈8–15 mT for $d_{\mathrm{Au}}>$ 2.5 nm is more than two times smaller when compared to recently reported values for YIG deposited on oxidized silicon ($\mu_{0}\Delta H_{0}=$ 31.8 mT) [44].

The obtained SW damping parameters for YIG/Au may enable coherent SW propagation in this system, which could be used to absorb SWs. To address this issue, we performed VNA spectroscopy measurements of SW transmission signals. Pairs of single-wire microwave antennas with a width of 2 μm were fabricated by photolithography and VNA spectroscopy measurements were conducted on YIG films with $d_{\mathrm{Au}}=$ 3, 3.5, and 4 nm, corresponding to $\mu_{0}\Delta H_{0}=$ 8.1, 8.0, and 10.7 mT, respectively. The distance between the exciting and detecting antennae was 20 μm.

Figure 2e shows the spectra for YIG/Au (3 nm) bilayer taken in the Damon-Eshbach geometry. First, we observe an absence of phase oscillations in the S_12_ signal indicating no SW propagation between excitation and detection antennas [28,57,58]. Instead, we see broad FMR spectra, similar to the S_22_ absorption displayed in Figure 2d. The detected signal can be interpreted as a distant induction of FMR via long-range stray fields [59]. The r.f. currents inductively generated in the receiving antenna further interact with the bilayer and produce changes in the S_12_ transmission signal. The effect is reproducible for Au thicknesses $d_{\mathrm{Au}}=$ 3.5 and 4 nm and correlates with the FMR intensity and linewidth (see additional data in the supplementary material). Angular dependence of the S_12_ spectra (Figure 2f) further points to the lack of SW propagation and additionally shows a small uniaxial anisotropy field of 2.0 ± 0.5 mT for the YIG/Au bilayer.

The S_12_ transmission spectra for YIG/Au bilayer allowed us to rule out the possibility of coherent SW propagation with large SW wavenumbers, i.e., corresponding to frequencies above the induced FMR signal. However, depending on the relative interplay between SW and inducted FMR signals, the oscillatory character of the SW phase on propagation may be hidden for long SWs [59]. To further verify this phenomenon, we have conducted SNS-MOKE measurements to investigate low-k excitations. As shown in Figure 2g, the intensity of SNS-MOKE signal quickly drops to zero over a distance of 2 μm. In addition, the line scans in Figure 2h show no oscillations for the out-of-plane magnetization component, proving that coherent SWs are not propagating in the bilayer. According to the measurements, we conclude that although the determined intrinsic damping for YIG/Au is relatively low (α≈ 8·10^−4^), the SW decay length is very short. This can be better understood by considering the effective damping $\alpha_{\mathrm{eff}}$ parameter encompassing both α and $\Delta H_{0}$ parameters:

(3) $$\alpha_{\mathrm{eff}}=\frac{\Delta f}{2f}\approx \left(\alpha +\frac{\gamma \mu_{0}\Delta H_{0}}{4\pi f}\right)\sqrt{1+{\left(\frac{\gamma \mu_{0}M_{\mathrm{eff}}}{4\pi f}\right)}^{2}}.$$

Equation (3) is derived for an in-plane applied magnetic field (see the supplementary material) and highlights the importance of the inhomogeneous linewidth broadening $\Delta H_{0}$ on SW damping. For a 3-nm-thick Au layer, the YIG film exhibits $\mu_{0}\Delta H_{0}\approx$ 8 mT. This gives the effective damping parameter $\alpha_{\mathrm{eff}}$ of 0.09 at 2 GHz, which is large and leads to strong SW damping. Furthermore, the insertion of a 1-nm-thick Pt or Au layer between YIG and GGG already increases the $\alpha_{\mathrm{eff}}$ parameter to ≈0.02–0.03 at 2 GHz (see inset in Figure 2c). Based on these results, we infer that thin metal underlayers could be used to absorb SWs in YIG waveguides without detrimental back reflection.

### 3.3. SW Packet Propagation Characteristics

Before we discuss micromagnetic simulations, we introduce the basic properties of SW packet propagation. The envelope |ψ(x,t)| of a SW packet can be described with:

(4) $$\left|\psi \left(x,t\right)\right|\propto A\left(k,t\right)\;e^{-\frac{1}{4}\;{\left(\frac{x-x_{0}-\omega^{\prime }\left(k\right)\;t}{\sigma \left(t\right)}\right)}^{2}},$$

in which, the amplitude A(k,t) is given by

(5) $$A\left(k,t\right)=\sqrt{\frac{\sigma_{x}}{\sqrt{\sigma^{2}\left(t\right)+\alpha_{\mathrm{eff}}\;\omega^{\prime \prime }\left(k\right)\;t}}}e^{-\alpha_{\mathrm{eff}}\;\omega \left(k\right)\;t},$$

and the SW packet broadening yields

(6) $$\sigma^{2}\left(t\right)=\sigma_{x}^{2}+{\left(\frac{\omega^{\prime \prime }\left(k\right)}{2\sigma_{x}}t\right)}^{2}.$$

Here, $\alpha_{\mathrm{eff}}$ is the effective damping parameter, $\sigma_{x}$ is the spatial width of the packet, ω(k) is the angular frequency depending on the wavenumber k, and $\omega^{\prime }\left(k\right)$ and $\omega^{\prime \prime }\left(k\right)$ are the first and the second derivative of ω(k), respectively. The derivation of Equation (4) is presented in the supplementary material. From the exponent in Equation (4), we can see that the pulse peak travels at the group velocity $\omega^{\prime }\left(k\right)$ and the packet broadens in time as described by σ(t).

The decay length $L_{d}$ of the propagating SW packet can thus be calculated from

(7) $$A\left(k,\tau \right)=\frac{1}{e},$$

with relaxation time $\tau =L_{d}/\omega^{\prime }\left(k\right)$. In the limiting cases of Equation (7), when $\omega^{\prime \prime }\left(k\right)=0$ or $\sigma_{x}\to \infty$, the amplitude $A\left(k,\tau \right)=e^{-\alpha_{\mathrm{eff}}\;\omega \left(k\right)\;\tau }$, so that the attenuation length yields:

(8) $$L_{d}^{\sigma_{x}\to \infty }=\frac{\omega^{\prime }\left(k\right)}{\alpha_{\mathrm{eff}}\;\omega \left(k\right)}.$$

If the spatial pulse width $\sigma_{x}\to 0$ and $\omega^{\prime \prime }\left(k\right)\neq 0$, the amplitude of the wave packet approaches zero. From an application point of view, it is, therefore, crucial to design SW devices so that the second derivative of the dispersion relation $\omega^{\prime \prime }\left(k\right)$ is minimized or, ideally, equal to zero to avoid amplitude loss and the SW packet broadening over time.

**Figure 3:**
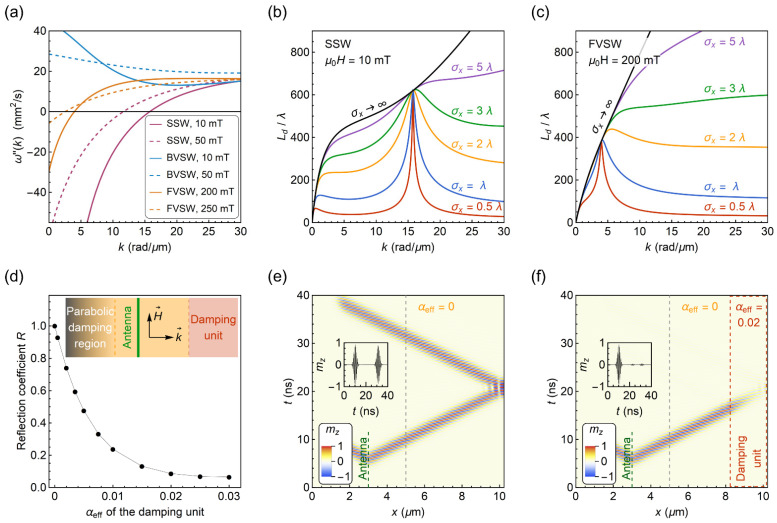
(**a**) Second derivative of the dispersion relation $\omega^{\prime \prime }\left(k\right)$ for the surface and backward volume, and the forward volume SWs. In (**b**, **c**), the ratio of the decay length $L_{d}$ to the wavelength λ is shown for SSW and FVSW, respectively, for different values of the SW packet spatial width $\sigma_{x}$. In figures (**a**–**c**), the dependencies are calculated for typical parameters of a 50-nm-thick epitaxial YIG film with saturation magnetization $M_{s}=$ 140 kA/m, exchange stiffness $D_{\mathrm{ex}}=$ 5.3·10^−17^ T∙m^2^, and effective damping $\alpha_{\mathrm{eff}}=$ 1·10^−4^. (**d**) Reflection coefficient as a function of the effective damping parameter $\alpha_{\mathrm{eff}}$ in the damping unit. The inset illustrates the simulation geometry. In (**e**,**f**), the time evolution of SW packet is shown for a damping unit with $\alpha_{\mathrm{eff}}=$ 0 and $\alpha_{\mathrm{eff}}=$ 0.02, respectively. The insets show time dependences of the SW amplitude taken at x= 5 μm (marked with grey dashed lines). Figures (**e**,**f**) are also visualized in supplementary Videos S1 and S2.

Analyzing the SW dispersion relations with an exchange term [60,61] for typical parameters of epitaxial YIG film (Figure 3a), we find that the condition $\omega^{\prime \prime }\left(k\right)=0$ can be met for surface spin-waves (SSW, or Damon-Eshbach modes) as well as for forward volume spin-waves (FVSW). However, the condition is not met for the backward volume spin-waves (BVSW) propagating in a continuous film when the external magnetic field is greater than zero. Moreover, the wavenumber k for which $\omega^{\prime \prime }\left(k\right)=0$ can be tuned by the external magnetic field for SSW and FVSW, as shown in Figure 3a. An increase of the magnetic field shifts the solution of $\omega^{\prime \prime }\left(k\right)=0$ toward lower wavenumbers k.

Following Equation (7), the decay length of SW packets is significantly decreased when $\omega^{\prime \prime }\left(k\right)\neq 0$ (Figure 3b,c). To counteract this effect, one could consider the use of longer excitation pulses at the expense of a decreasing density of information that can be encoded and the speed of computation. Therefore, a careful choice of k (or equivalently, excitation frequency at a given bias field) is vital for the design of SW conduits to closely match the $\omega^{\prime \prime }\left(k\right)=0$ condition.

### 3.4. SW Packet Reflection from a Damping Unit

To validate the application of the bilayers as an SW absorber, we performed micromagnetic simulations in *MuMax3* [41] to study the reflection of a SW packet from an area with different values of the effective damping parameter $\alpha_{\mathrm{eff}}$. For the modeling, we treat YIG/metal bilayer as an effective medium described with $\alpha_{\mathrm{eff}}$, and therefore, neglect the microstructural properties of the bilayer. The simulation geometry is schematically shown in the inset in Figure 3d. It consists of an antenna excitation region at x= 3 μm, an area with enhanced effective magnetic damping at 8 μm <x< 10 μm, and an artificial damping region at 0 μm <x< 2 μm, where parabolic damping conditions are defined to avoid SW reflection from the left edge. The YIG film is discretized into 1024 × 32 × 1 cells with a size of 10 × 10 × 50 nm^3^ and one-dimensional periodic boundary conditions are applied along the *y*-axis to mimic a continuous film. The parameters for the YIG layer are: saturation magnetization $M_{s}=$ 140 kA/m, exchange stiffness [62] $D_{\mathrm{ex}}=$ 5.3·10^−17^ T∙m^2^, and YIG film thickness $d_{\mathrm{YIG}}=$ 50 nm. The Gilbert damping parameter for 2 μm <x< 8 μm is set to zero and $\alpha_{\mathrm{eff}}$ is varied for the damping area. To excite the wave packet, we used a Gaussian-enveloped sinusoidal magnetic pulse. A frequency of 1.7 GHz at the external bias field $\mu_{0}H=$ 10 mT was optimized in order to minimize wave packet dispersion for the surface waves, as discussed above. For these conditions, the spatial width of the pulse is $\sigma_{x}=$ 0.41 μm, which is comparable to the SW wavelength λ= 0.69 μm and the width of the damping area. We recorded the time evolution of the out-of-plane component of magnetization $m_{z}$ for 40 ns.

To evaluate the SW reflection, we compared the amplitude of the reflected wave packet to the incident one:

(9) $$R=\frac{A_{r}}{A_{i}},$$

where the amplitudes $A_{r}$ and $A_{i}$ are obtained by the Fourier transform of the reflected and incident signal at x= 5 μm. As shown in Figure 3e, when $\alpha_{\mathrm{eff}}$ for the damping unit is equal to zero as for the rest of the film, the SW packet is fully reflected from the right edge so that R= 1. One can also clearly see that the propagating wave packet does not disperse in time, i.e., the SW packet broadening and the associated amplitude loss is not observed over time. For an increasing $\alpha_{\mathrm{eff}}$ parameter, we find that R decreases gradually (Figure 3d). At $\alpha_{\mathrm{eff}}=$ 0.02 (also visualized in Figure 3f), the reflection coefficient yields R= 0.086, and for $\alpha_{\mathrm{eff}}=$ 0.03, R= 0.065. The decrease in amplitude of the reflected signal, which is more than an order of magnitude, substantiates the potential application of the YIG/metal bilayers as an efficient SW absorber. Moreover, $\alpha_{\mathrm{eff}}\approx$ 0.02–0.03 can already be met for a very thin metal underlayer of ≈1 nm for Pt or Au as calculated with Equation (3) (see inset in Figure 2c). Additionally, the insertion of such a thin metal layer does not deteriorate the effective magnetization significantly which further strongly supports the low reflection coefficients.

## 4. Summary

In conclusion, we investigated the structural and magnetic properties of YIG films grown onto GGG substrates with thin metal layers. The insertion of nanometer-thick platinum, iridium, or gold films at the YIG/GGG interface enables accurate tuning of the effective magnetic damping through material selection and variation of the metal underlayer thickness. Micromagnetic simulations based on parameters derived from experiments show that YIG/Au or YIG/Pt bilayers can be used as effective SW absorbers that can limit the reflection coefficient of SWs to R < 0.1. Applications of such absorbers are envisioned in integrated magnonic circuits.

## Data Availability

The data presented in this study are available on request from the corresponding author.

## Supplementary Materials

The following supporting information can be downloaded at: https://www.mdpi.com/article/10.3390/ma15082814/s1, X-ray reflectometry results; Gi-XRD measurements of YIG/Ir/GGG (111); AFM additional data; VNA-FMR basic data; VNA spectroscopy results for YIG/Au/GGG (111); Derivation of spin-wave packet evolution in a dispersive medium; Calculation of the effective damping parameter; Spin wave dispersion relations; Fourier transform of the magnetic pulse and the spin-wave packet; Basic material properties of bulk Ir, Pt, Au, YIG and GGG [48,63,64,65,66]; Video S1: Total spin-wave packet reflection from the film edge; Video S2: Spin-wave packet absorption by the damping unit.
