# Supplementary material for: Tuning of Magnetic Damping in Y3Fe5O12/Metal Bilayers for Spin-Wave Conduit Termination
Source: Materials (Basel). 2022 Apr 12;15(8):2814. doi: 10.3390/ma15082814 (PMC9030244; doi:10.3390/ma15082814)
Supplement: Supplementary file 1 [file materials-15-02814-s001.zip › Supplementary Information.pdf]

Supplementary Information

Tuning of Magnetic Damping in $Y_3Fe_5O_{12}$ / Metal Bilayers for Spin-Wave Conduit Termination

Adam Krysztofik[1*], Nikolai Kuznetsov[2], Huajun Qin[2], Lukáš Flajšman[2], Emerson Coy[3], and Sebastiaan van Dijken[2]

[1] *Institute of Molecular Physics, Polish Academy of Sciences, ul. Smoluchowskiego 17, 60-179 Poznań, Poland*

[2] *NanoSpin, Department of Applied Physics, Aalto University School of Science, P.O. Box 15100, FI-00076 Aalto, Finland*

[3] *NanoBioMedical Centre, Adam Mickiewicz University, ul. Wszechnicy Piastowskiej 3, 61-614 Poznań, Poland*

* adam.krysztofik@ifmpan.poznan.pl

- X-ray reflectometry results
- Gi-XRD measurements of YIG / Ir / GGG (111)
- AFM additional data
- VNA-FMR basic data
- VNA spectroscopy results for YIG / Au / GGG (111)
- Spin-wave packet evolution in a dispersive medium
- Calculation of the effective damping parameter
- Spin wave dispersion relations
- Fourier transform of the magnetic pulse and the spin-wave packet
- Basic material properties of bulk Ir, Pt, Au, YIG and GGG

X-ray reflectometry results

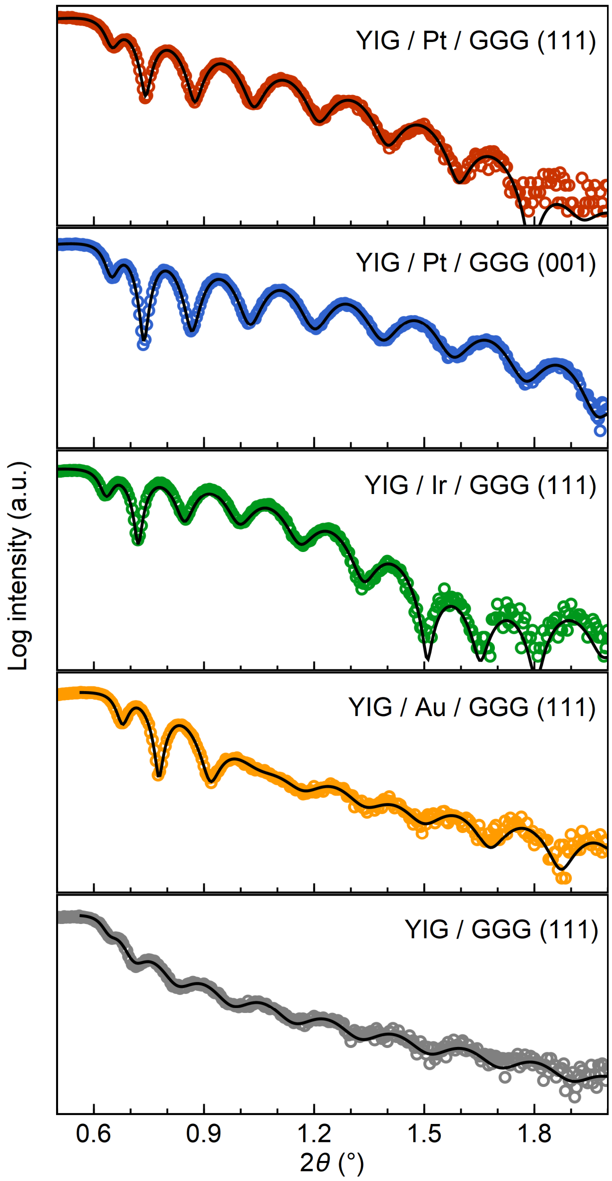

The XRR measurements were performed using an additional vertical slit limiting incident X-ray radiation to 1.5 mm across the metal wedge which corresponds to a change in metal thickness by 0.75 nm. The determined thickness of YIG films ranges between 38.9 and 42.4 nm pointing to good stability of deposition conditions. The roughness of YIG layers grown on metal spacers is increased to 1.0-1.5 nm in comparison to the roughness of the YIG layer grown directly on the GGG substrate (0.7 nm). The determined critical angle of YIG films on top of metal spacers ranges from 0.602° to 0.640°, in congruence with 0.618° for a YIG/GGG reference measurement, and therefore suggests a consistent density of YIG layers.

Gi-XRD measurements of YIG / Ir / GGG (111)

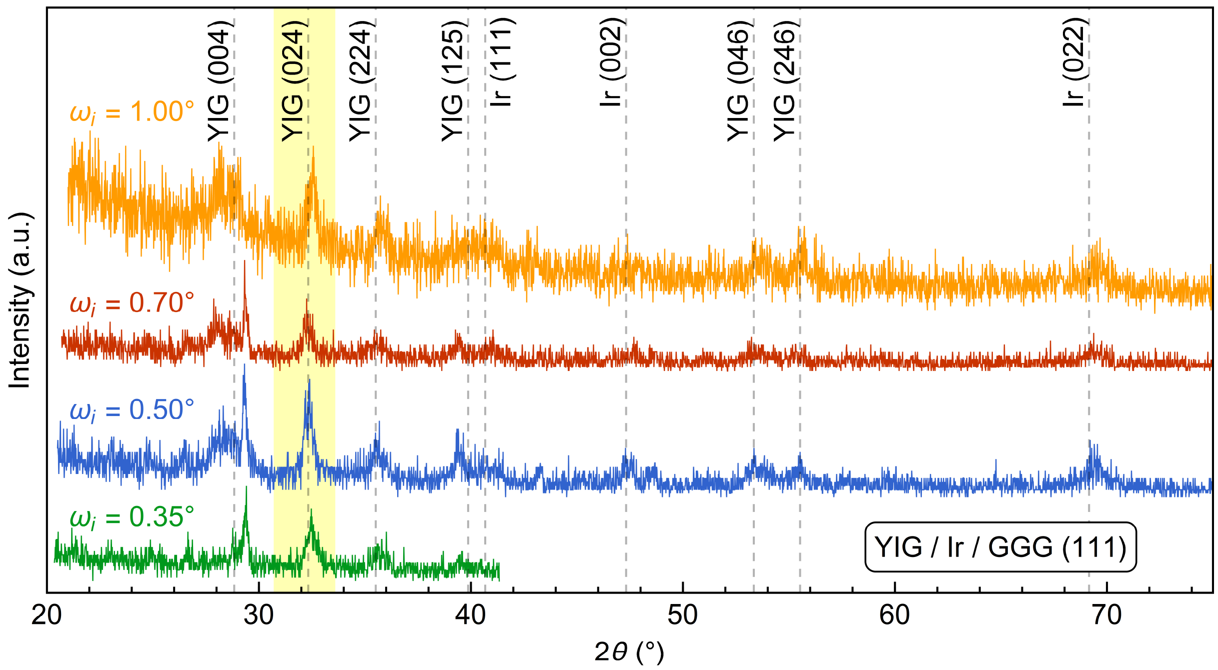

Grazing incident X-ray diffraction for YIG / Ir / GGG (111) sample. The measurements were taken for incident angles ω_i between 0.35-1.00° and show the polycrystalline structure of both films. Dashed lines mark reflection positions for bulk YIG and Ir [42, 45]. Analyzing the width of the highest intensity reflection (024) for YIG with the Scherrer formula, the estimated crystallite size yields 16.9 ± 2.7 nm as averaged from these four scans. The determined lattice parameter of YIG is equal to 1.235 ± 0.004 nm in agreement with the bulk value (1.2375 nm) [42].

AFM additional data

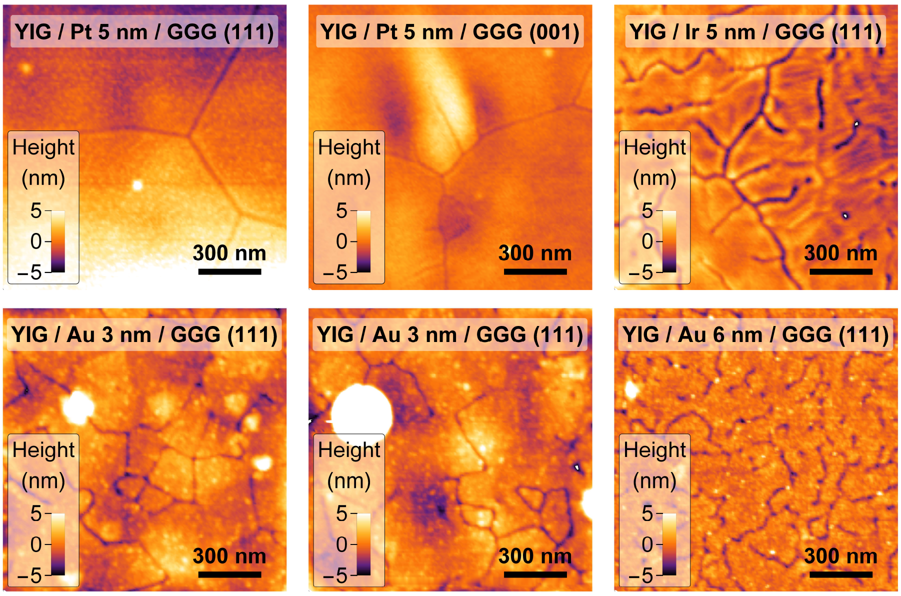

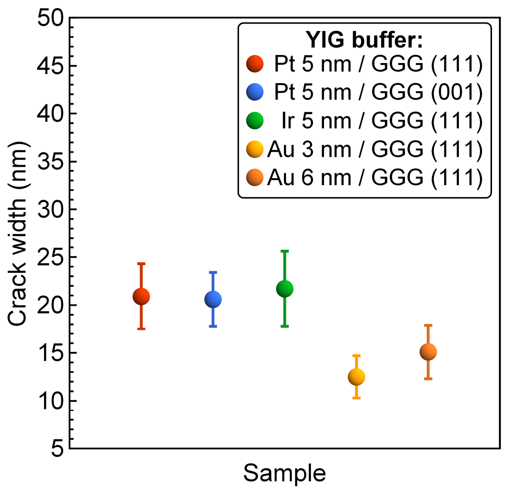

Estimation of crack width based on AFM surface imaging indicating a maximal separation distance between parts of the YIG film. Error bars denote the standard deviation of the mean represented by points. The statistics is based on 30 crack measurements for each sample.

VNA-FMR basic data

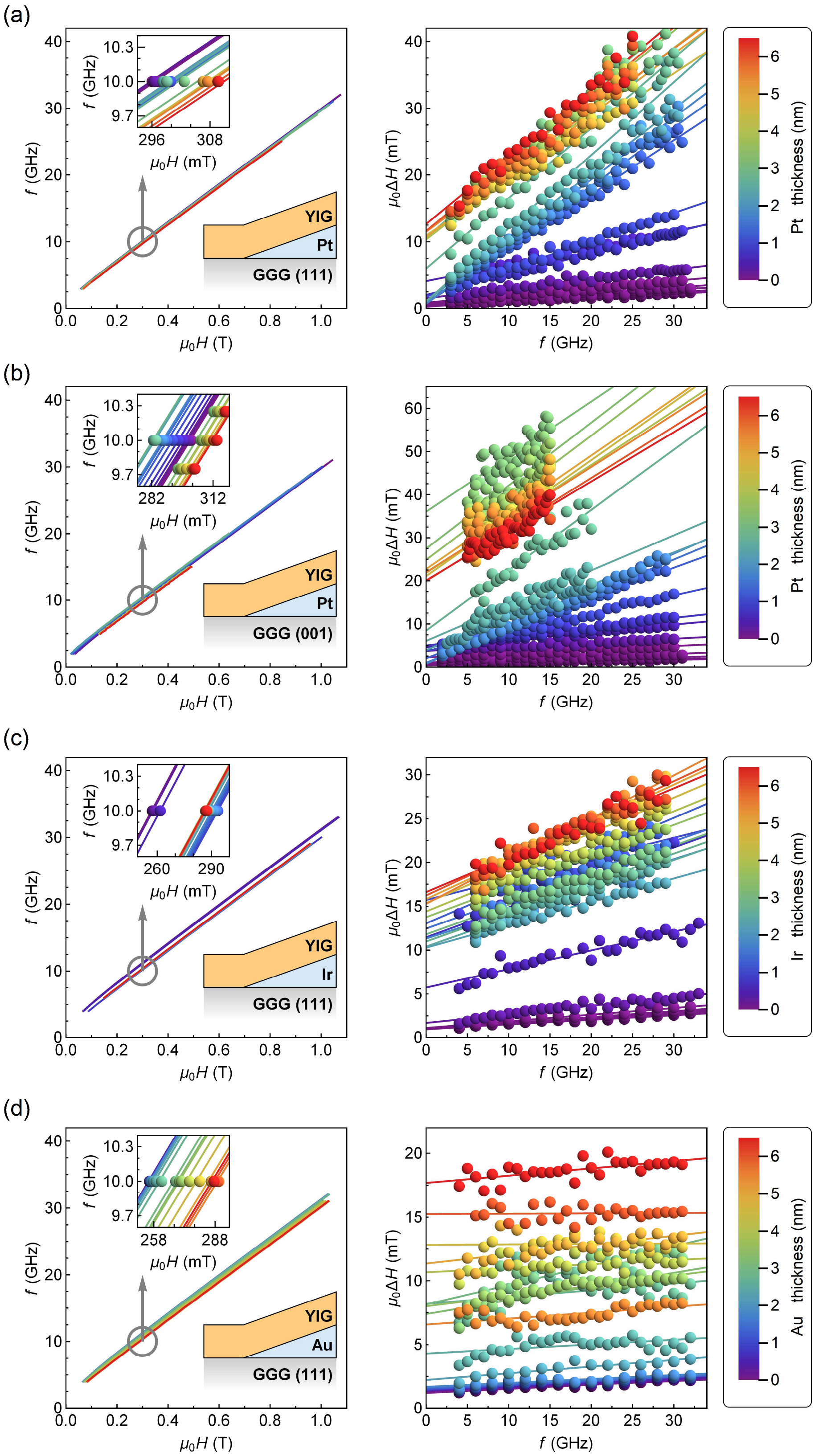

Kittel relation and linewidth dependence on frequency for: (a) YIG/Pt/GGG (111), (b) YIG/Pt/GGG (001), (c) YIG/Ir/GGG (111), and (d) YIG/Au/GGG (111) samples.

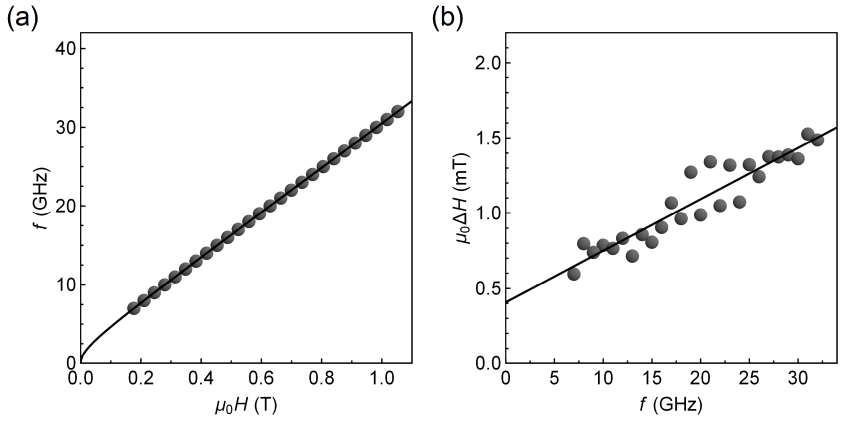

(a) Kittel relation and (b) linewidth dependence on frequency for the epitaxial YIG grown on GGG substrate. The sample was prepared directly in the pulsed laser deposition chamber and have not been in any contact with the magnetron plasma. From the fittings we estimate the effective magnetization M_{eff} =137.5 ± 0.1 kA/m, the Gilbert damping parameter α =(4.8 ± 0.4)·10^{-4}, and the inhomogeneous linewidth broadening $\mu_0\Delta H_0$ =0.41 ± 0.05 mT.

VNA spectroscopy results for YIG / Au / GGG (111)

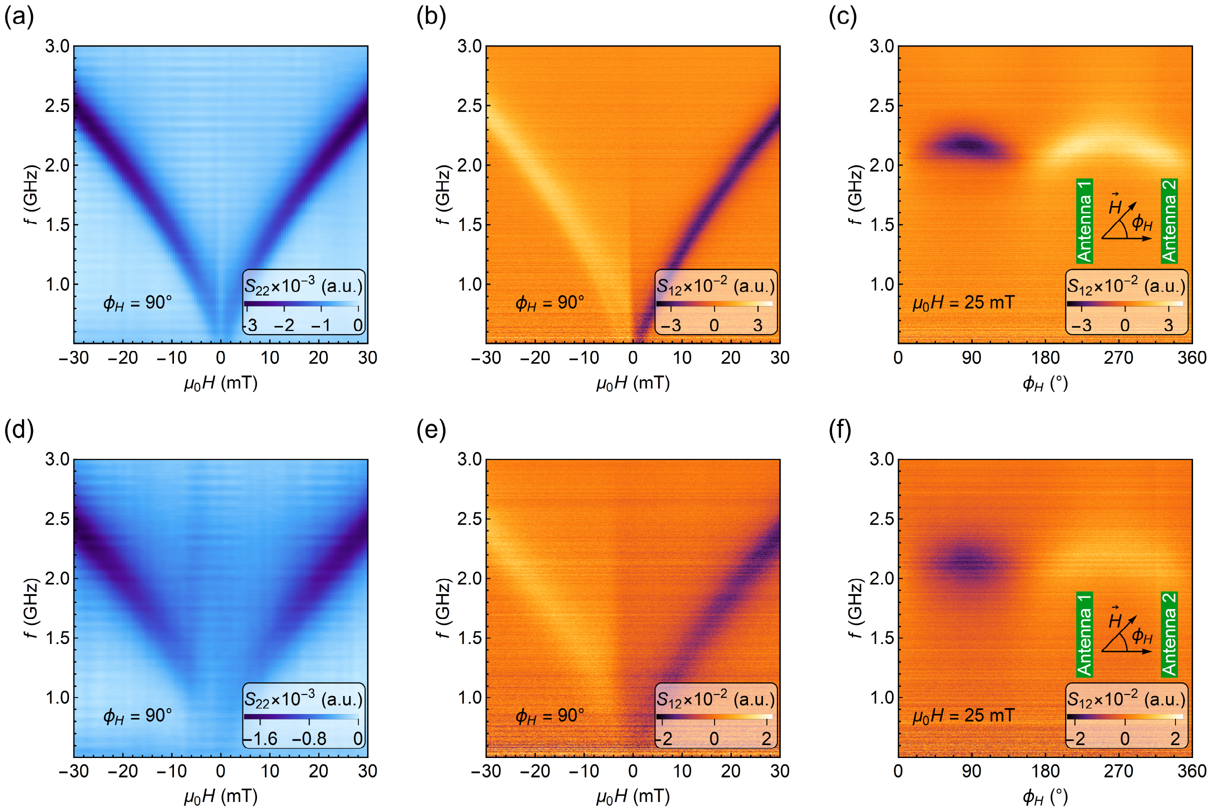

VNA spectroscopy results measured with lithographically patterned antennas for (**a-c**) YIG (40 nm) / Au (3.5 nm) / GGG (111), and (**d-f**) YIG (40 nm) / Au (4 nm) / GGG (111). (**a,d**) Color-coded reflection parameter S_{22} showing the FMR absorption. (**b,e**) Color-coded transmission parameter S_{12} for the magnetic field aligned parallel to the antennas ($\phi_H = 90°$). (**c,f**) Color-coded angular dependence of S_{12} spectrum at $\mu_0 H = 25$ mT. Inset depicts in-plane magnetic field orientation with respect to the antenna geometry. In all figures, the real part of the scattering parameter S_{pq} is plotted.

Spin-wave packet evolution in a dispersive medium

The wavefunction can be calculated using the inverse Fourier transformation:

$$\psi(x,t) \propto \int_{-\infty}^{\infty} \overline{\psi}(k) e^{i(kx-\omega(k)t)} dk, \tag{S1}$$

where $\overline{\psi}(k)$ is derived from

$$\overline{\psi}(k) \propto \int_{-\infty}^{\infty} \psi(x, t=0) e^{-ikx} dx, \tag{S2}$$

and the pulse at time $t = 0$ with carrier wavenumber k_c and the spatial width σ_x has a form:

$$\psi(x, t=0) \propto e^{-\frac{(x-x_0)^2}{4\sigma_x^2}} e^{ik_c x}. \tag{S3}$$

Here, we truncate the dispersion relation to the second-order term $(k-k_c)^2$ in Taylor expansion

$$\omega(k) = \omega(k_c) + (k-k_c)\omega'(k_c) + \frac{1}{2}(k-k_c)^2\omega''(k_c), \tag{S4}$$

and find that the integral in Eq. (S1) yields

$$\psi(x,t) \propto \frac{1}{\sqrt{1+i\omega''(k_c)t/(2\sigma_x^2)}} e^{-\left(\frac{x-x_0-\omega'(k_c)t}{2\sigma(t)}\right)^2} e^{i\phi(x,t)}, \tag{S5}$$

$$\phi(x,t) = k_c x - \omega(k_c)t + \frac{\omega''(k_c)t}{2\omega''^2(k_c)t^2+8\sigma_x^4}(x - x_0 - \omega'(k_c)t)^2, \tag{S6}$$

$$\sigma^2(t) = \sigma_x^2 + \left(\frac{\omega''(k_c)}{2\sigma_x} t\right)^2. \tag{S7}$$

The $e^{i\phi(x,t)}$ component of the solution describes only wave oscillations. It is however worth noting that $\omega''(k_c) \neq 0$ in Eq. S6 is responsible for an additional modulation of the oscillations.

By taking the absolute value $|\psi(x,t)|$ we can identify the signal envelope and better read off the temporal evolution of the wave packet:

$$|\psi(x,t)| \propto \sqrt{\frac{\sigma_x}{\sigma(t)} e^{-\frac{1}{2}\left(\frac{x-x_0-\omega'(k_c)t}{\sigma(t)}\right)^2}}. \tag{S8}$$

The exponent in Eq. (S8) shows that the wave packet travels at the group velocity $\omega'(k_c)$ and it broadens in time. Concurrently, the amplitude $\sqrt{\sigma_x/\sigma(t)}$ of the signal decreases. As can be inferred from $\sigma(t)$ (Eq. (S7)), both the pulse broadening and the amplitude drop result from $\omega''(k_c) \neq 0$. Hence, it is important to extend the analysis to account for intrinsic magnetic losses related to the choice of a spin-wave medium.

By introducing a decay term $e^{-\alpha_{\text{eff}}\omega(k)t}$ into Eq. (S1), where α_{eff} is the effective damping parameter and repeating the calculations, we find:

$$|\psi(x,t)| \propto \sqrt{\frac{\sigma_x}{\sqrt{\sigma^2(t)+\alpha_{\text{eff}}\,\omega''(k_c)t}}\, e^{-2\alpha_{\text{eff}}\,\omega(k_c)t} e^{-\frac{1}{2}\left(\frac{x-x_0-\omega'(k_c)t}{\sigma(t)}\right)^2}}. \quad \text{(S9)}$$

Hence, we can calculate the decay length of propagating spin-wave packet by numerically solving the following equation:

$$\frac{1}{e} = \sqrt{\frac{\sigma_x}{\sqrt{\sigma^2(\tau)+\alpha_{\text{eff}}\,\omega''(k_c)\tau}}}\, e^{-\alpha_{\text{eff}}\,\omega(k_c)\tau}, \quad \text{(S10)}$$

with relaxation time $\tau = L_d/\omega'(k_c)$. The decay length L_d is defined here as the distance at which the amplitude of the wave packet has been reduced by a factor of e.

Calculation of the effective damping parameter

The relation between the relaxation time τ and the spectral width Δω can be found using the Fourier transform of a damped oscillator:

$$\tilde{A}(\omega) = \int_0^{\infty} A(t) e^{-i\omega t}\, dt, \quad \text{(S11)}$$

$$A(t) = e^{-\frac{t}{\tau}}\, e^{i\omega_0 t}. \quad \text{(S12)}$$

Calculating integral in Eq. S11, one obtains the real part of $\tilde{A}(\omega)$ as a Lorentz function:

$$\text{Re}\left[\tilde{A}(\omega)\right] = \frac{\frac{1}{\tau}}{\left(\frac{1}{\tau}\right)^2 + (\omega-\omega_0)^2}, \quad \text{(S13)}$$

so that the relation between τ and Δω is given by:

$$\frac{1}{\tau} = \frac{\Delta\omega}{2}. \quad \text{(S14)}$$

Hence, the relaxation term in Eq. S12 can be written in terms of an effective damping parameter α_{eff}:

$$e^{-\frac{t}{\tau}} = e^{-\alpha_{\text{eff}}\omega t}, \quad \text{(S15)}$$

by using

$$\alpha_{\text{eff}} = \frac{\Delta\omega}{2\omega}. \quad \text{(S16)}$$

As can be seen from Eq. S16, the frequency-swept FMR experiment, in which $\Delta\omega = 2\pi\Delta f$, provides, therefore, direct information on relaxation process and can be used for evaluation of the effective damping straightforwardly. However, in the field-swept ferromagnetic resonance, the FWHM linewidth ΔH is described with the Heinrich formula:

$$\mu_0\Delta H = \frac{2\alpha}{\gamma}\omega + \mu_0\Delta H_0, \tag{S17}$$

where α is the intrinsic Gilbert damping parameter, and ΔH_0 is the inhomogeneous linewidth broadening. The required transition to the frequency domain can be well approximated with:

$$\Delta\omega = \frac{\partial\omega}{\partial H}\Delta H. \tag{S18}$$

Combining equations S16-S18, the effective damping parameter now yields:

$$\alpha_{\mathrm{eff}} = \left(\alpha + \frac{\gamma\mu_0\Delta H_0}{2\omega}\right)\frac{1}{\gamma\mu_0}\frac{\partial\omega}{\partial H}. \tag{S19}$$

An explicit form of Eq. S19 can be found calculating the derivative $\frac{\partial\omega}{\partial H}$ from Kittel equations. For an in-plane applied magnetic field H_{IP}, for which

$$\omega = \mu_0\gamma\sqrt{H_{\mathrm{IP}}(H_{\mathrm{IP}} + M_{\mathrm{eff}})}, \tag{S20}$$

and $\omega = 2\pi f$, one obtains:

$$\alpha_{\mathrm{eff}}^{\mathrm{IP}} = \left(\alpha + \frac{\gamma\mu_0\Delta H_0}{4\pi f}\right)\sqrt{1 + \left(\frac{\gamma\mu_0 M_{\mathrm{eff}}}{4\pi f}\right)^2}, \tag{S21}$$

which provides accurate numerical estimates for $f > 1$ GHz. For the out-of-plane applied field H_{OP}, when

$$\omega = \mu_0\gamma(H_{\mathrm{OP}} - M_{\mathrm{eff}}), \tag{S22}$$

one finds:

$$\alpha_{\mathrm{eff}}^{\mathrm{OP}} = \alpha + \frac{\gamma\mu_0\Delta H_0}{4\pi f}. \tag{S23}$$

Both equations S21 and S23 clearly show the relevance of ΔH_0 parameter on magnetization relaxation.

Spin wave dispersion relations

For the calculations presented in Fig. 3 (a-c) the following spin-wave dispersion relations have been used [60, 61]:

- surface spin-waves (SSW), film magnetized in-plane, $\vec{k}\perp\vec{M}$:

$$\omega(k) = \gamma\sqrt{(\mu_0 H + D_{\text{ex}}k^2)(\mu_0 H + D_{\text{ex}}k^2 + \mu_0 M_s) + \left(\frac{1}{2}\mu_0 M_s\right)^2 (1 - e^{-2kd})}, \quad \text{(S24)}$$

- backward volume spin-waves (BVSW), film magnetized in-plane, $\vec{k} \parallel \vec{M}$:

$$\omega(k) = \gamma\sqrt{(\mu_0 H + D_{\text{ex}}k^2)\left(\mu_0 H + D_{\text{ex}}k^2 + \mu_0 M_s \frac{1-e^{-kd}}{kd}\right)}, \quad \text{(S25)}$$

- forward volume spin-waves (FVSW), film magnetized out-of-plane, $\vec{k}\perp\vec{M}$:

$$\omega(k) = \gamma\sqrt{(\mu_0 H + D_{\text{ex}}k^2 - \mu_0 M_s)\left(\mu_0 H + D_{\text{ex}}k^2 - \mu_0 M_s \frac{1-e^{-kd}}{kd}\right)}, \quad \text{(S26)}$$

where $\omega = 2\pi f$ is the angular frequency, γ is the gyromagnetic ratio, μ_0 is the vacuum permeability, k is the wavenumber, H is the bias magnetic field, M_s is the saturation magnetization, D_{ex} is the exchange stiffness, d is the film thickness. Within Eq. S24-S26, the group velocity $\omega'(k) = \frac{\partial\omega}{\partial k}$ and the second derivative $\omega''(k) = \frac{\partial^2\omega}{\partial k^2}$ have been calculated.

Fourier transform of the magnetic pulse and the spin-wave packet

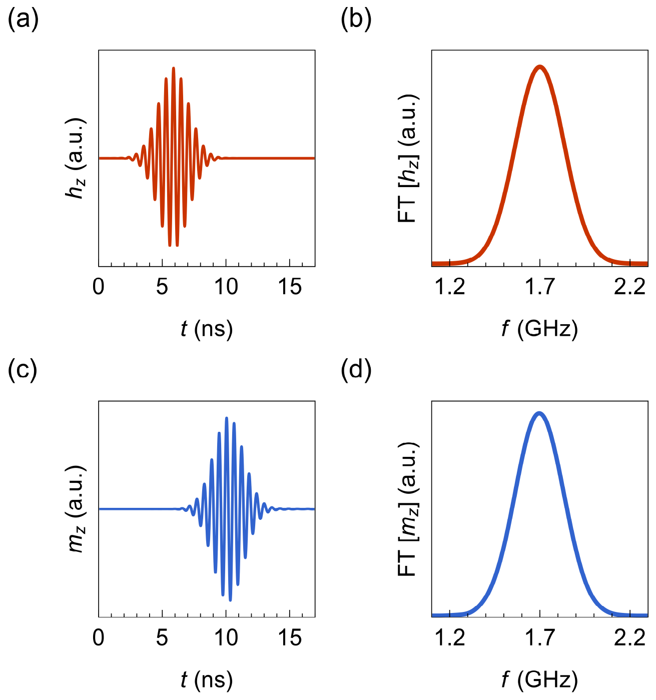

(a) Gaussian-enveloped sinusoidal magnetic pulse (z-component, h_z) used for spin-wave packet excitation and (b) its Fourier transform FT $[h_z]$. (c) Propagating spin-wave packet (z-component, m_z) and (d) its Fourier transform FT $[m_z]$.

Basic material properties of bulk Ir, Pt, Au, YIG and GGG

**Material**	**Atomic number**	**Melting point (°C)**	**Thermal expansion coefficient ($\times 10^{-6}$ K^{-1})**	**Tensile strength (MPa)**	**Plasticity**
Iridium	77	2450	6.4	500-1000	Brittle
Platinum	78	1770	8.8	117	Ductile
Gold	79	1060	14.2	138	Ductile
YIG	–	1555	9.9-11.4	–	Brittle
GGG	–	1800	8.3-8.7	–	Brittle

Data was taken from Ref. [48, 63-66].
